# Short-Term Outcomes of Micropulse Transscleral Cyclophotocoagulation Using the VITRA 810 and Postoperative Ciliochoroidal Effusion in Japanese Patients

**DOI:** 10.3390/jcm14217453

**Published:** 2025-10-22

**Authors:** So Handa, Takahiro Kawaji, Shin-ichi Otsuka

**Affiliations:** 1 Otsuka Eye Clinic, 3-12-69 Tanaka-machi, Oita 870-0852, Japan; 2 Sato Eye and Internal Medicine Clinic, 4160-270 Arao, Kumamoto 864-0041, Japan; kawag@white.plala.or.jp

**Keywords:** glaucoma, micropulse transscleral cyclophotocoagulation, MP-TSCPC, VITRA 810, Japan, ciliochoroidal effusion, phthisis bulbi

## Abstract

**Background**: This study aimed to evaluate the short-term outcomes of micropulse transscleral cyclophotocoagulation (MP-TSCPC) using the VITRA 810 in Japanese patients, focusing on intraocular pressure (IOP), medication score, complications, and ciliochoroidal effusion (CE). **Methods**: We retrospectively reviewed 37 eyes of 34 patients treated between March 2024 and March 2025. Postoperative IOP, glaucoma medication score, complications, and CE assessed by anterior segment OCT were analyzed. IOP changes were compared between CE-positive and CE-negative groups. **Results**: The mean preoperative IOP was 22.8 ± 7.1 mmHg, which decreased to 13.3 ± 6.8 mmHg at 1 week, 14.9 ± 7.4 mmHg at 1 month, 14.9 ± 5.8 mmHg at 3 months, and 15.0 ± 5.2 mmHg at 6 months (all *p* < 0.05 vs. baseline). CE was observed in 22 eyes (59.4%) and was associated with greater IOP reduction at 1 week and 1 month. Complications included mydriasis (24.3%), anterior chamber inflammation (24.3%), cystoid macular edema (5.4%), decreased visual acuity (8.1%), and phthisis bulbi (2.7%). **Conclusions**: VITRA 810 MP-TSCPC achieved significant short-term IOP reduction in Japanese patients. CE was linked to greater early IOP lowering. While generally safe, rare but severe complications such as phthisis bulbi require close monitoring.

## 1. Introduction

Glaucoma is a group of progressive optic neuropathies characterized by retinal ganglion cell loss and optic nerve damage and is one of the leading causes of irreversible blindness worldwide [1,2]. In Japan, glaucoma accounts for approximately 40% of newly certified cases of visual disability, underscoring its major public health impact [3]. Lowering intraocular pressure (IOP) remains the only evidence-based strategy to slow or prevent disease progression across glaucoma subtypes [2,4]. Consequently, there is sustained interest in treatments that achieve consistent IOP reduction while minimizing treatment burden and procedure-related risk.

Transscleral cyclophotocoagulation (TSCPC) has traditionally been reserved for refractory disease with limited visual potential because continuous-wave (CW) diode cyclophotocoagulation can be associated with serious adverse events, including persistent hypotony and phthisis bulbi [5]. In parallel with a shift toward earlier, less invasive interventions—catalyzed by evidence supporting selective laser trabeculoplasty (SLT) as a primary option and by the growth of microinvasive glaucoma surgery (MIGS) [6]—micropulse transscleral cyclophotocoagulation (MP-TSCPC) has emerged as a potentially safer cyclodestructive modality. MP-TSCPC delivers 810 nm infrared energy in an on–off duty cycle (31.3%), which limits collateral thermal damage and improves procedural reproducibility relative to CW approaches [7]. Typical CW-TSCPC is applied spot-by-spot at ~2000 mW for ~2 s per application, titrated to an audible “pop,” whereas—per consensus recommendations—MP-TSCPC is commonly performed at ~2500 mW with a 31.3% duty cycle while sweeping the probe over the superior and inferior hemispheres for a total of ~160 s; parameters may vary by institution [8]. Since its introduction on the CYCLO G6 platform in 2015, multiple studies have reported clinically meaningful IOP reductions with acceptable safety across disease severities [7,8,9,10,11,12,13,14,15,16,17,18,19].

The VITRA 810, introduced in Japan in April 2024, differs from the CYCLO G6 in that it originated as a multicolor retinal photocoagulation system with an added micropulse transscleral glaucoma module. Because probe design and energy-delivery characteristics can influence tissue coupling and dose distribution, platform-specific data are needed—particularly in Asian populations, where ocular pigmentation may affect laser–tissue interactions. However, real-world evidence for the VITRA 810 remains limited.

The mechanisms underlying IOP reduction after MP-TSCPC appear multifactorial, including subthreshold effects on the ciliary epithelium that reduce aqueous production, ciliary muscle changes that enhance trabecular outflow, and extracellular matrix remodeling near the pars plana that facilitates uveoscleral outflow [20,21,22,23,24,25,26,27]. Anterior segment optical coherence tomography (AS-OCT) enables in vivo detection of postoperative ciliochoroidal effusion (CE), which has been reported after MP-TSCPC and may reflect enhanced uveoscleral outflow [28].

Against this backdrop, we conducted a single-center, retrospective study to characterize short-term outcomes of MP-TSCPC performed with the VITRA 810 in Japanese patients. Our objectives were to: (i) quantify 6-month IOP changes, medication use, and safety events; and (ii) determine the frequency of AS-OCT–detected CE and test whether the presence of CE is associated with greater early IOP lowering.

## 2. Materials and Methods

### 2.1. Study Design and Patients

This retrospective study included 37 eyes from 34 consecutive patients with glaucoma who underwent MP-TSCPC using the VITRA 810 between March 2024 and March 2025 at Otsuka Eye Clinic, Oita, Japan. The study was conducted in accordance with the Declaration of Helsinki and was approved by the Institutional Review Board of Otsuka Eye Clinic (approval code: 202402). Written informed consent was obtained from all participants before surgery. Eligible eyes had open-angle glaucoma without active inflammation, including primary open-angle glaucoma (POAG), pseudoexfoliation glaucoma (PXG), and secondary open-angle glaucoma without active inflammation. Exclusion criteria were secondary glaucoma with active inflammation, intraocular surgery within the previous 3 months, and follow-up of less than 1 month after MP-TSCPC.

### 2.2. Surgical Technique

MP-TSCPC was performed by two surgeons (S.H.: first author, S.O.: senior author). Sub-Tenon anesthesia was administered using 2% lidocaine.

The transscleral probe of the VITRA 810 was applied to the superior and inferior hemispheres of the sclera, approximately 3 mm posterior to the limbus, avoiding the 3- and 9-o’clock positions as well as any preexisting filtering blebs or implant sites.

Laser parameters were set at a power of 2000–2500 mW, with a maximum exposure time of 80 s per hemisphere and a duty cycle of 31.3%, according to the surgeon’s discretion. The probe was moved in a continuous sweeping manner during irradiation. Postoperatively, topical gatifloxacin (0.3%) and betamethasone (0.1%) were instilled four times daily for at least one week. Glaucoma medications prescribed before surgery were generally maintained throughout the follow-up period to allow evaluation of the surgical effect of MP-TSCPC itself.

### 2.3. Data Collection and Statistical Analysis

All patients underwent baseline screening, which included slit-lamp biomicroscopy, fundus examination, refraction testing, IOP measurement (iCare IC200; M.E. Technica Co., Tokyo, Japan), and BCVA assessment using a Landolt C chart. To minimize diurnal variation, IOP was measured at approximately the same time in the morning at each visit whenever feasible. Refraction was measured with an autorefractor/keratometer (ARK-530A; Nidek Co., Tokyo, Japan). BCVA values were converted to the logarithm of the minimum angle of resolution (logMAR). Postoperatively, all patients were evaluated at 1 week, 1 month, 3 months, and 6 months. The glaucoma medication score was calculated by assigning one point for each single-agent medication and two points for each fixed-combination preparation. At each postoperative visit, IOP, medication score, and complications were assessed. Endothelial cell density was measured at 1 month using a specular microscope (CE-530; Nidek Co., Tokyo, Japan). The presence of ciliochoroidal effusion (CE) was evaluated using AS-OCT (CASIA2; TOMEY Corp., Aichi, Japan) at the 3-, 6-, 9-, and 12-o’clock meridians at 1 week and 1 month postoperatively, and AS-OCT was performed in all cases at 1 week after MP-TSCPC and repeated until CE became negative. CE was defined as a continuous hyporeflective space between the ciliary body and the sclera; eyes were classified as CE(+) if this finding was present at any of the 3-, 6-, 9-, or 12-o’clock meridians. AS-OCT assessments were performed by a single examiner experienced in grading. Preoperative visual field testing was performed with the Humphrey Field Analyzer (Carl Zeiss Meditec, Dublin, CA, USA) using the SITA-Standard 24-2 protocol. Patients rated ocular pain immediately after surgery using a 0–10 visual analog scale (VAS). The primary outcome was the change in IOP from baseline. Secondary outcomes included changes in glaucoma medication score, the incidence of postoperative complications, the presence of CE, and surgical success. Surgical success was defined as an IOP between 6 and 21 mmHg or a reduction of at least 20% from baseline without additional glaucoma surgery. Failure was defined as an IOP less than 6 mmHg, greater than 21 mmHg despite maximal therapy, or the need for reoperation. Analyses were conducted in JMP 14 (SAS Institute Inc., Cary, NC, USA). Pre–post comparisons used paired *t*-tests; CE(+) vs. CE(−) comparisons used Wilcoxon rank-sum tests. Predictors of CE were assessed with logistic regression, and surgical success was summarized with Kaplan–Meier methods. Two-sided *p* < 0.05 was considered statistically significant. We additionally fit linear mixed-effects models with patient ID as a random intercept and baseline IOP as a covariate to compare IOP at 1 week and 1 month by CE status.

## 3. Results

### 3.1. Overall Outcomes

**Figure 1:**
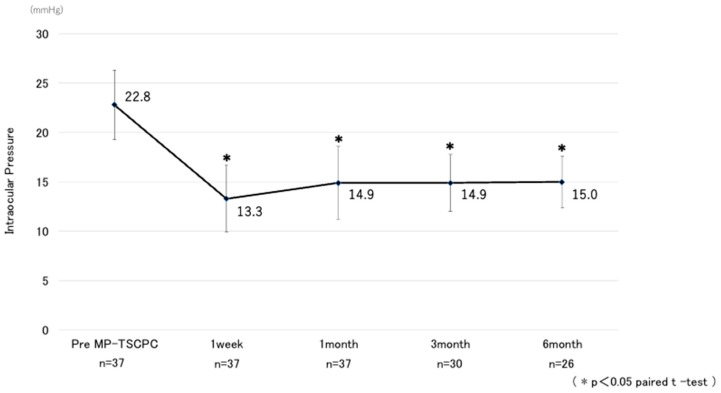
Changes in mean IOP before and after MP-TSCPC using the VITRA 810. Error bars represent standard deviation. Asterisks indicate statistically significant reductions compared with baseline (paired *t*-test, *p* < 0.05).

**Figure 2:**
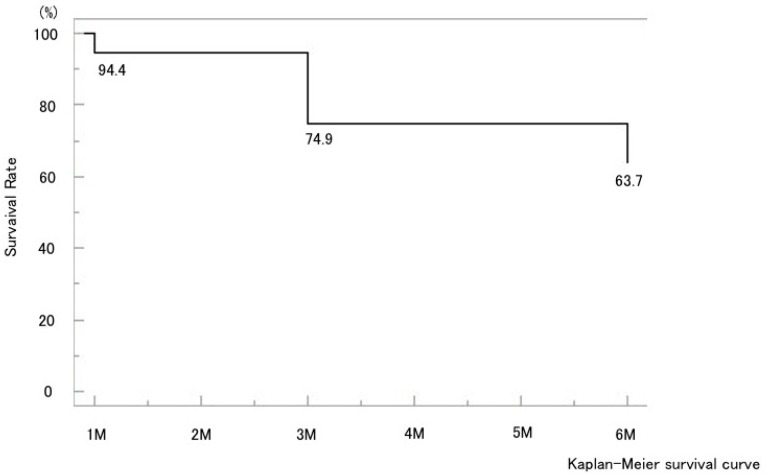
Kaplan–Meier survival curves for surgical success after MP-TSCPC using the VITRA 810. Overall cumulative probability of success, with rates of 94.4% at 1 month, 74.9% at 3 months, and 63.7% at 6 months.

**Figure 3:**
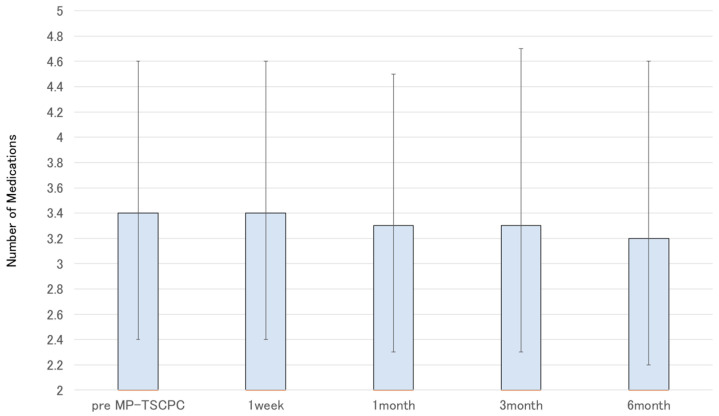
Changes in the mean number of glaucoma medications before and after MP-TSCPC using the VITRA 810. The mean number of medications was 3.4 preoperatively, 3.4 at 1 week, 3.3 at 1 month, 3.3 at 3 months, and 3.2 at 6 months. The slight reduction observed over time did not reach statistical significance.

A total of 37 eyes from 34 patients were analyzed. The baseline preoperative characteristics of the patients are summarized in Table 1. The mean preoperative IOP was 22.8 ± 7.1 mmHg and showed significant decreases at all postoperative time points, measuring 13.3 ± 6.8 mmHg at one week, 14.9 ± 7.4 mmHg at one month, 14.9 ± 5.8 mmHg at three months, and 15.0 ± 5.2 mmHg at six months, with all reductions significant compared with baseline (*p* < 0.05) (Figure 1). The mean IOP reduction rate at six months was 34.2%. Kaplan–Meier analysis demonstrated a cumulative probability of surgical success of 63.7% at six months (Figure 2). The glaucoma medication score decreased slightly by six months but without statistical significance (Figure 3).

### 3.2. Comparison Between CE(+) and CE(−) Groups

**Figure 4:**
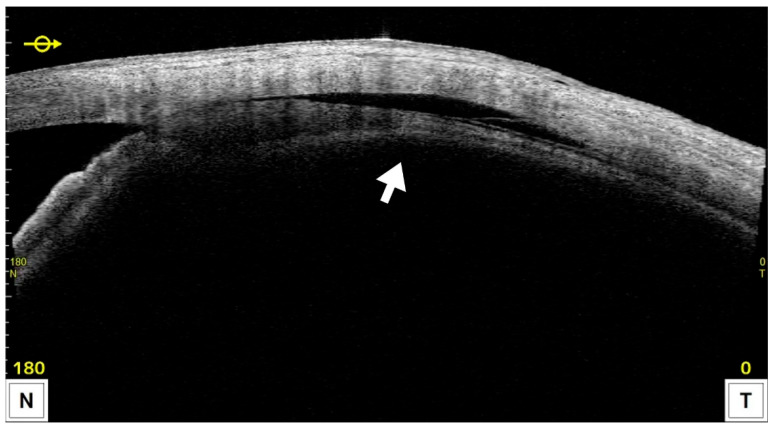
Representative AS-OCT image showing ciliochoroidal effusion (CE) after MP-TSCPC using the VITRA 810. The hyporeflective area between the ciliary body and sclera (arrow) indicates the presence of CE.

**Figure 5:**
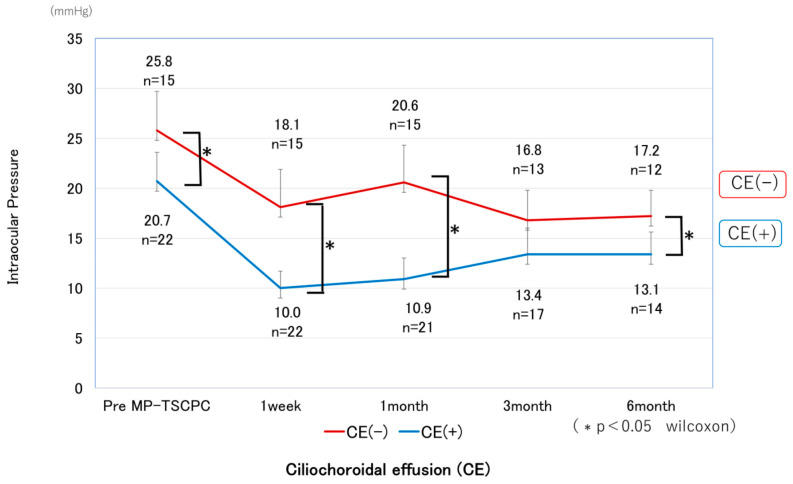
Comparison of IOP changes between CE(+) and CE(−) eyes after MP-TSCPC with the VITRA 810. The CE(+) group showed a significantly greater reduction at 1 week, 1 month, and 6 months (*p* < 0.05). Error bars indicate standard deviation, and asterisks denote significant differences (Wilcoxon test, *p* < 0.05).

**Figure 6:**
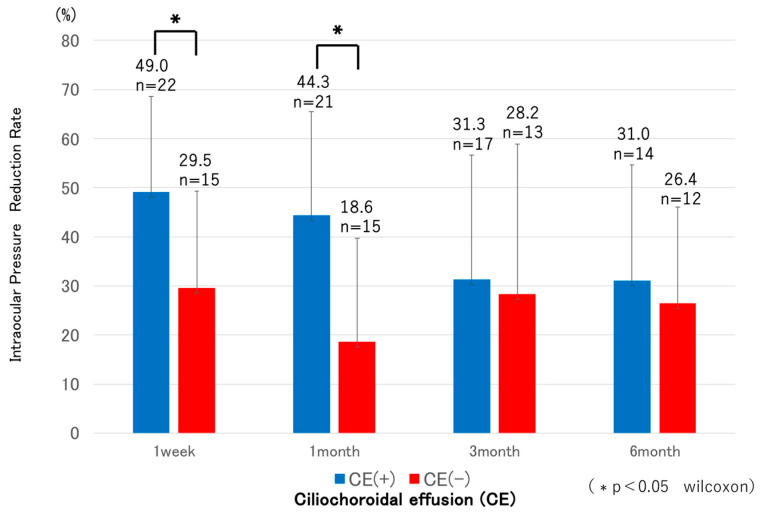
Comparison of IOP reduction rates between eyes with and without CE. The CE(+) group showed significantly greater reductions at 1 week and 1 month (*p* < 0.05), while no differences were observed at 3 and 6 months. Asterisks indicate significant differences (Wilcoxon test, *p* < 0.05).

**Figure 7:**
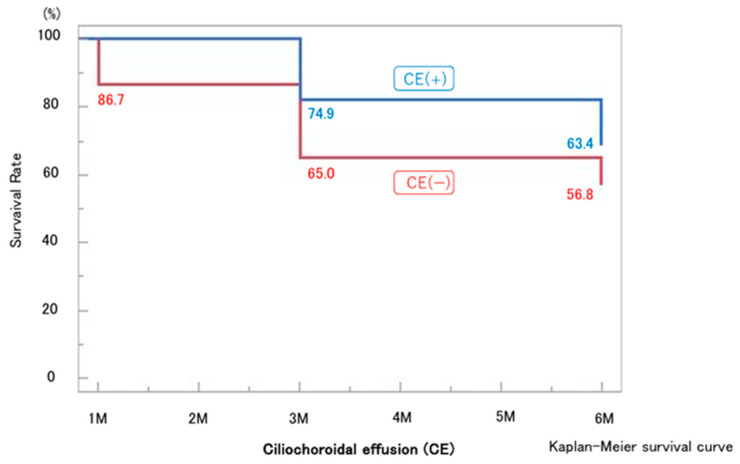
Kaplan–Meier survival analysis showing no significant difference in 6-month survival between the CE(+) group (63.4%) and the CE(−) group (56.8%) (*p* = 0.32).

CE was detected by AS-OCT (Figure 4) in 22 eyes (59.4%) at 1 week and had resolved in all cases by 1 month postoperatively, except for one eye that developed phthisis bulbi; thereafter, IOP in the CE-positive group tended to increase. In most CE(+) cases, CE was observed in one or two quadrants at 1 week postoperatively; however, in the case that developed phthisis bulbi, marked CE was present in all four quadrants even at 3 months after MP-TSCPC. Both groups showed significant IOP reductions from baseline at all postoperative time points (*p* < 0.05). In the CE(+) group, IOP decreased from 20.7 ± 5.8 mmHg preoperatively to 10.0–13.4 mmHg during follow-up (31–49% reduction). In the CE(−) group, IOP decreased from 25.8 ± 7.8 mmHg to 16.8–20.6 mmHg (18–29% reduction). Between-group comparisons showed significantly lower IOP in the CE(+) group at baseline and at 1 week, 1 month, and 6 months (Figure 5), while the reduction rates were significantly greater in the CE(+) group at 1 week and 1 month (*p* < 0.05; Figure 6). The overall 6-month survival rate was 63.7% (Figure 2); rates were 63.4% in CE(+) and 56.8% in CE(−), with no significant difference (*p* = 0.32; Figure 7).

In a mixed-effects model with patient ID as a random intercept and baseline IOP as a covariate, eyes with CE had significantly lower IOP at 1 week (fixed effect of CE: F = 7.54, *p* = 0.0105). The adjusted least-squares means were 16.65 mmHg for CE(−) and 11.54 mmHg for CE(+), indicating an approximately 5.1-mmHg lower IOP in the CE(+) group. In the same framework, eyes with CE at 1 week also had significantly lower IOP at 1 month (fixed effect of CE: F = 21.24, *p* < 0.0001), corresponding to an adjusted difference of ~4.82 mmHg (higher in CE(−) vs. CE(+)). Baseline IOP was significantly associated with week-1 IOP (F = 11.14, *p* = 0.0022) but not with 1-month IOP (*p* = 0.945). No statistically significant associations were found between CE presence and sex, age, glaucoma subtype, prior surgery, baseline IOP, or baseline visual field status (*p* > 0.05).

### 3.3. Postoperative Complications

Best-corrected visual acuity did not change significantly overall, although three eyes (8.1%) lost at least 0.3 logMAR units. Endothelial cell density remained stable at one month. Postoperative complications included mydriasis, anterior chamber inflammation, hyphema, cystoid macular edema, decreased visual acuity, and phthisis bulbi, and are summarized in Table 2. The mean postoperative pain score on the VAS was 3.6 ± 3.3.

## 4. Discussion

Evidence on MP-TSCPC with the VITRA 810 remains limited, as most prior studies have used the CYCLO G6 platform. We evaluated the clinical efficacy and safety of VITRA 810 MP-TSCPC in a Japanese cohort and examined the association between ciliochoroidal effusion (CE) and intraocular pressure (IOP) outcomes. In all cases, the mean IOP decreased significantly from 22.8 ± 7.1 mmHg preoperatively to 15.0 ± 5.2 mmHg at 6 months postoperatively, corresponding to a mean reduction rate of 34.2%. Previous studies using the CYCLO G6 have reported IOP reductions of approximately 30–45% [9,10,11,12], which is consistent with our findings. The survival rate, defined as an IOP between 6 and 21 mmHg or a ≥20% reduction from baseline without reoperation, was 63.7% at 6 months. Prior reports using a similar definition [10,11,13] described survival rates ranging from 45.5% to 83.3%, and our results fall within this range.

Pain during MP-TSCPC was reported as 3.6 ± 3.3 on a 10-point visual analog scale. As MP-TSCPC is inherently associated with ocular discomfort, adequate anesthesia is essential. Retrobulbar or sub-Tenon anesthesia is frequently employed, but these methods can cause transient visual impairment; therefore, special caution is required when performing MP-TSCPC in the only seeing eye in an outpatient setting.

The most common postoperative complication was mydriasis, observed in 9 eyes (24.3%). This may result from injury to the terminal branches of the short ciliary nerves. Chen et al. reported an incidence of 3.3%, and Radhakrishnan et al. reported 11%, with particularly higher rates in Asian patients (odds ratio 13.07) [10,14]. Although most cases resolve spontaneously, some patients require pilocarpine therapy to alleviate photophobia. In this study, six patients were treated with 1% pilocarpine for symptomatic photophobia, which was discontinued after approximately one month when symptoms improved. Two eyes still exhibited incomplete recovery of pupil size at the final follow-up, though with a trend toward resolution.

Anterior chamber inflammation is a commonly reported complication, with incidences ranging from a few percent to 25% [10,15,16]. We observed it in 24.3% of cases, supporting the use of postoperative anti-inflammatory eye drops. One case of hyphema occurred in an eye with a prior trabeculotomy, likely due to reflux bleeding following postoperative IOP reduction. Cystoid macular edema (CME), though uncommon (several to 5% of cases [16,17,18]), developed in two eyes. In one patient with a history of uveitis, CME was likely triggered by postoperative inflammation and resolved promptly with sub-Tenon’s injection of triamcinolone acetonide.

Three eyes experienced a loss of more than 0.3 logMAR in visual acuity. Among the three eyes that showed decreased visual acuity, two had severe central visual field defects preoperatively, with only a small residual central field despite relatively good visual acuity. After MP-TSCPC, intraocular pressure decreased; however, visual acuity declined without any other apparent complications that could explain the decrease, suggesting further progression of the preexisting visual field loss. The remaining eye with decreased visual acuity was the case that developed phthisis bulbi. Although cataract progression has been reported as a cause of visual decline after MP-TSCPC [19], it was not observed in our study. Persistent hypotony and phthisis bulbi reported elsewhere have been attributed to excessive energy delivery or suboptimal surgical technique [16]. In our case, although severe anterior segment inflammation was noted postoperatively, total delivered energy (125.2 J) was within the recommended range for the CYCLO G6 (P3 probe) [20]. Because the VITRA 810 probe tip is narrower than that of the CYCLO G6, it is possible that improper angulation toward the cornea could result in unintended irradiation of the ciliary processes rather than the pars plana. Furthermore, given that eyes with darker pigmentation generally respond to lower laser energy [29,30,31], the energy level used may have been excessive for this patient. The occurrence of complications such as anterior chamber inflammation and cystoid macular edema may also have been related to excessive laser energy. Therefore, further studies are warranted to establish optimal energy parameters suitable for Asian populations when using the VITRA 810.

In this study, CE was assessed using AS-OCT (CASIA2) at 1 week and 1 month. CE was observed in 22 eyes (59.4%) at 1 week, and eyes in the CE-positive group exhibited significantly greater IOP reduction and reduction rates compared with CE-negative eyes at both 1 week and 1 month. CE had resolved by 1 month in all eyes except the one that developed phthisis bulbi, after which IOP in the CE-positive group demonstrated a trend toward an increase. In mixed-effects models that accounted for within-patient clustering and adjusted for baseline IOP, the presence of CE remained an independent predictor of lower IOP at 1 week and 1 month (adjusted differences ≈ 5.1 and 4.8 mmHg, respectively). Notably, although a higher baseline IOP is typically associated with larger percentage reductions after pressure-lowering interventions, eyes with CE exhibited lower baseline IOP yet achieved greater early IOP reductions. This inversion of the expected gradient strengthens the inference that CE itself augments the early pressure-lowering response—either as an effect modifier or as part of the treatment mechanism. The temporal pattern—CE detected at 1 week predicting lower IOP at 1 month—further supports a causal pathway. A plausible biological explanation is that CE reflects enhanced uveoscleral outflow, in line with prior histologic and imaging observations of ciliary muscle bundle widening and increased choroidal thickness after MP-TSCPC. Clinically, AS-OCT–detected CE may serve as an early on-treatment biomarker of response. The association was not evident at later time points, suggesting that CE-mediated effects are transient and that other mechanisms (e.g., subthreshold effects on ciliary epithelium) may sustain longer-term IOP control. These findings warrant prospective confirmation with pre-specified mixed-model analyses and quantitative outflow imaging.

The mechanisms of IOP reduction by MP-TSCPC are thought to be multifactorial, including subthreshold damage to pigmented and non-pigmented ciliary epithelium leading to reduced aqueous humor production [21,22,23], pilocarpine-like contraction of the ciliary muscle enhancing trabecular outflow [24,25,26], and extracellular matrix remodeling near the pars plana promoting uveoscleral outflow [27,28]. CE may indicate an enhancement of uveoscleral outflow, supported by pathological findings of widened ciliary muscle bundles and OCT evidence of increased choroidal thickness in human eyes [25,27]. Consistent with our findings, Chansangpetch et al. reported that CE-positive eyes had significantly greater IOP reduction at 1 month post-MP-TSCPC [29]. In the present study, a more pronounced reduction in IOP was also observed in the CE-positive group, suggesting that CE may reflect enhanced uveoscleral outflow.

Future prospective studies incorporating quantitative analyses of aqueous humor dynamics using OCT or UBM, as well as histopathological evaluations, are warranted.

The VITRA 810 has the potential to expand the indications of CPC beyond refractory cases, contributing to earlier intervention and avoidance of repeat surgery.

In particular, it may serve as a valuable minimally invasive treatment option for patients who decline incisional glaucoma surgery, such as filtering procedures, or for those with poor systemic conditions that make such surgeries difficult.

This study has limitations. First, although we modeled within-patient clustering using mixed-effects analyses with patient ID as a random intercept, residual correlation or heterogeneity between fellow eyes cannot be completely excluded. Second, the retrospective, single-center design with a modest sample size and 6-month follow-up may limit generalizability and preclude firm conclusions about long-term efficacy and safety. Third, laser energy parameters were set at the surgeons’ discretion, which introduces treatment-protocol variability and potential confounding. Fourth, AS-OCT was obtained at 1 week and 1 month only, CE was defined qualitatively by a single masked grader, and quantitative outflow measures were not available. Fifth, given the exploratory scope, *p*-values were not adjusted for multiple comparisons and should be interpreted as hypothesis-generating. Finally, there was no concurrent control group (e.g., CYCLO G6), so platform-level comparisons are inferential rather than causal.

## 5. Conclusions

MP-TSCPC using the VITRA 810 significantly reduced IOP in Japanese patients with glaucoma in the short term. Postoperative CE was frequently observed and associated with greater early IOP reduction, suggesting a possible mechanism of enhanced uveoscleral outflow. Although overall safety was acceptable, rare severe complications such as phthisis bulbi emphasize the importance of close monitoring. These results provide new evidence on the performance of the VITRA 810 in Japan, and larger, longer-term studies are required to confirm these findings.

## Figures and Tables

**Table 1:** Baseline characteristics of included patients.

Parameter	Value
Number of eyes, n	37
Age, years	79.3 ± 9.5
Male/Female, n	18/19
BCVA, logMAR	0.4 ± 0.5
Visual field MD, dB	−18.8 ± 9.7
IOP, mmHg	22.8 ± 7.1
Number of medications	3.4 ± 1.2
Endothelial cell density (ECD), cells/mm^2^	2014.2 ± 718.2
Intraocular lens eyes/Phakic eyes, n	32/5
Number of previous glaucoma surgeries	1.2 ± 1.1
Type of previous glaucoma surgeries, n	
Selective laser trabeculoplasty	11
MP-TSCPC	13
Conventional trabeculotomy (ab externo)	2
Trabeculotomy (ab interno)	11
Trabeculectomy	3
Ex-PRESS implantation	3
Ahmed glaucoma valve implantation	1
Revision of trabeculectomy	1
Type of glaucoma, n	
−POAG	11
−PXG	22
−SOAG	4

Values are presented as mean ± standard deviation (SD) unless otherwise noted. BCVA, best-corrected visual acuity; MD, mean deviation; IOP, intraocular pressure; MP-TSCPC, micropulse transscleral cyclophotocoagulation; ECD, endothelial cell density; SD, standard deviation; POAG, primary open-angle glaucoma; PXG, pseudoexfoliation glaucoma; SOAG, secondary open-angle glaucoma.

**Table 2:** Postoperative complications.

Complication	No. of Eyes, n (%)
Mydriasis	9 (24.3%)
Anterior chamber inflammation	9 (24.3%)
Hyphema	1 (2.7%)
Cystoid macular edema	2 (5.4%)
Decreased visual acuity	3 (8.1%)
Phthisis bulbi	1 (2.7%)

Values are presented as the number of eyes (percentage).

## Data Availability

Data are available from the corresponding author on reasonable request.
